# Advancing mental health equity: a community-based adaptation of problem management plus (PM+) on a mobile health clinic

**DOI:** 10.3389/fpubh.2026.1800509

**Published:** 2026-06-19

**Authors:** Josephina Lin, Piper Derenoncourt, Rainelle Walker-White, Nancy Oriol, Daniel Palazuelos, Giuseppe Raviola, Stephanie L. Smith, Mollie Williams

**Affiliations:** 1 Harvard Medical School, Boston, MA, United States; 2 Department of Social and Behavioral Sciences, Harvard T.H. Chan School of Public Health, Boston, MA, United States; 3 Department of Global Health and Social Medicine, Harvard Medical School, Boston, MA, United States; 4 Beth Israel Deaconess Medical Center, Boston, MA, United States; 5 Brigham and Women’s Hospital, Boston, MA, United States; 6 Partners In Health, Boston, MA, United States; 7 Massachusetts General Hospital, Boston, MA, United States

**Keywords:** community health worker (CHW), community-based mental health care, global mental health, mental health equity, mobile health clinics, problem management plus (PM+), task-shifting, underserved communities

## Abstract

Mental health inequities persist in underserved communities due to systemic barriers to care. Healthy Roads, a mental wellness support program developed by the mobile clinic The Family Van, adapted the World Health Organization’s Problem Management Plus (PM+) curriculum to address these disparities with support from Partners In Health. PM+ is a brief, evidence-based psychological intervention designed for trained non-specialists to deliver in low-resource settings. This community case study describes the adaptation, implementation, and early lessons learned from delivering PM+ with trained community health workers (CHWs) among racially and linguistically diverse communities in Boston, Massachusetts. Data were collected from 49 participants who engaged in 173 sessions between January 2021 and December 2023. Quantitative outcomes were measured using the Psychological Outcomes Profiles (PSYCHLOPS) tool, alongside qualitative insights from session notes and client feedback. Participants who completed the program experienced a 25.4% reduction in mental health distress 95% CI [13.1, 37.9%], with those attending at least one session experiencing an 11.4% reduction 95% CI [5.8, 17.0%]. This program also demonstrates how community-based mental health initiatives can contribute to broader systems of collaborative learning where frontline providers, participants, community members, and institutional partners jointly adapt and refine program delivery to inform equitable adaptation and dissemination of mental health practices. As the first adaptation of PM + in a community-based mobile clinic in the United States, Healthy Roads offers implementation insights for community health programs on building a mental health workforce reflective of the community, centering community expertise, adapting program delivery for cultural relevance, and ensuring proper staffing to sustain quality care.

## 1 Introduction

Mental health and emotional wellbeing are central to public health in the United States, with depression and anxiety among the leading causes of years lived with disability (YLD) (1, 2). Despite recognition of the importance of mental wellbeing, progress in ensuring equitable access to supportive services has lagged behind other health concerns (3). Although evidence-based psychological treatments exist, there remains a need for accessible, effective care (4, 5). Gaps in access are particularly severe in communities of color, where structural and social barriers limit care (6, 7). These barriers contribute to persistent distress without timely or appropriate support, even in settings with high provider density.

Nationally, over half of adults experiencing mental illness do not receive treatment each year, with even greater gaps for low-income, immigrant, and racially marginalized populations. Only about 50.6% of U.S. adults with any mental illness received treatment, with substantially lower rates among Black, Hispanic, and Asian adults compared to White adults (8). Massachusetts and the U.S. also face a shortage of diverse mental health providers, exacerbating barriers to utilizing mental health services among historically marginalized communities (9). These inequities are also driven by cost, language barriers, limited culturally concordant care, stigma, and distrust of health systems shaped by structural racism.

To address the need for culturally relevant and accessible mental health services, The Family Van (TFV), a mobile clinic in Boston, Massachusetts, partnered with Partners In Health (PIH), a global non-profit health system strengthening organization, to develop Healthy Roads, an adaptation of The World Health Organization’s Problem Management Plus (PM+). PM+ is a low-intensity, cognitive-behavioral, problem-solving intervention for people experiencing chronic adversity with demonstrated effectiveness (10, 11). PM+ is designed for use by non-specialist providers, including lay providers, in settings with few mental health resources (12). This task-shifting model aligns with calls to diversify the mental health workforce and expand care delivery beyond licensed specialists, particularly in communities of color disproportionately impacted by provider shortages (13–15).

There is strong evidence supporting PM+ in low-and middle-income countries but limited literature about implementation within community-based, non-clinical settings in the United States (16–20). Emerging evidence suggests PM+ may also be effective in high-income settings, particularly for marginalized populations facing barriers to care (21–23). Evidence from recent studies underscores both the promise and complexity of implementing PM + in high-income countries, including the need for flexible delivery models, integration within stepped-care systems, and sustained organizational and policy support (23, 24). Additional implementation-focused research is needed in high-income settings to examine how task-shifting and community-based mental health delivery models can expand access, address workforce shortages, and improve cultural responsiveness.

This community case study aims to (1) describe the first adaptation and implementation of PM + within a community-based mobile health clinic, (2) examine early program outcomes using measures of psychological distress and qualitative reports of participant experience, and (3) identify key insights for delivering culturally responsive, non-specialist mental health support in underserved communities in the United States. In addition to examining early outcomes, this paper also highlights how community-based adaptation of mental health interventions can serve as a collaborative learning process in which learnings co-created and shared across participants, CHWs, staff, and community partners continuously refine care delivery over time and inform broader approaches to mental health support. In doing so, this paper contributes evidence on how community-driven mental health models can apply local knowledge to advance mental health equity.

## 2 Context

Boston faces substantial mental health inequities, with residents of predominantly Black and Latinx neighborhoods reporting significantly higher levels of frequent mental distress compared to their White counterparts (25). Statewide workforce shortages, high turnover among clinicians, long wait times, and a lack of racial and linguistic concordance between providers and patients disproportionately affect communities of color. In 2021, more mental health clinicians left community-based roles than were hired, further constraining access to care (26). This mismatch between high need and limited, culturally concordant care contributes to persistent unmet mental health needs and shaped the rationale for implementing Healthy Roads within a trusted, community setting. TFV’s longstanding presence in these communities positions the mobile clinic as a site of continuous, community-informed learning.

**Figure 1:**
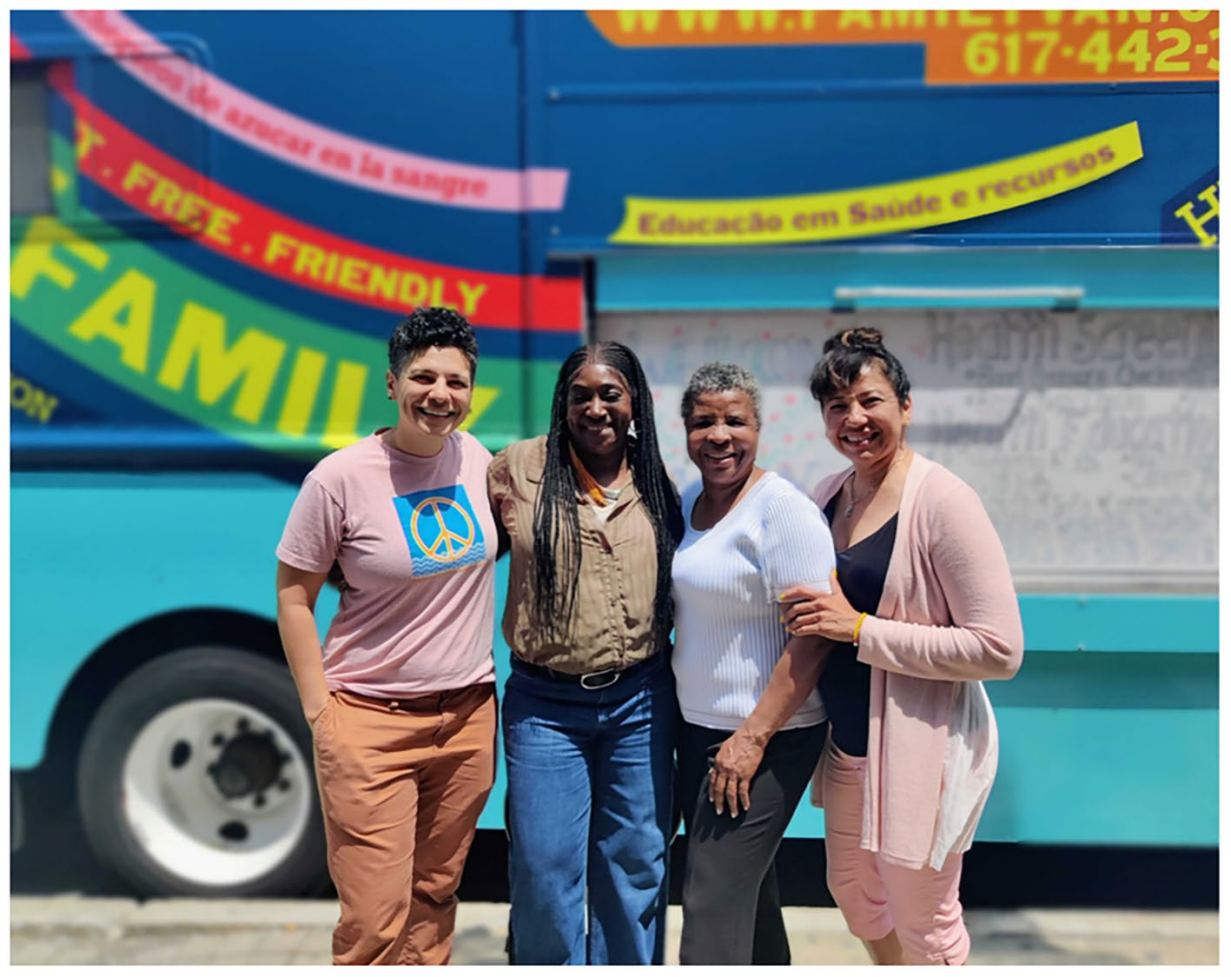
Members of the Family Van community health worker team at a community site in East Boston.

TFV is a mobile health clinic founded in 1992 that serves Boston communities with high levels of unmet health and social needs. Operated by community health workers who reflect the neighborhoods they serve, the van makes regular stops in Dorchester, Roxbury, and East Boston (Figure 1). TFV provides free preventive services including screenings, health coaching, education, and community referrals without appointments or insurance.

Ninety percent of clients identify as people of color, 57% speak a language other than English, and 50% are immigrants. Most have at least one chronic health condition and face compounding social stressors, including food insecurity, housing instability, and racism. Community members report that these structural and social stressors are inseparable from mental health distress yet rarely addressed in traditional mental health care.

A 2018 community assessment by TFV identified structural barriers to mental health services including limited access; provider-patient discordance in race, ethnicity, and language; long wait-times; and insurance and immigration-related barriers (27). These findings are consistent with CHW observations and underscore the need for low-barrier, culturally grounded mental health support embedded within trusted community services rather than siloed specialty care. This bidirectional exchange between community knowledge and program implementation shaped the adaptation of PM+ within the local context. The authors are aware of two other implementations of PM+ in the United States and no published evaluations. This gap supports the importance of documenting a community-based, mobile adaptation of PM+ in Boston.

## 3 Key programmatic elements

Healthy Roads is a community-based adaptation of the World Health Organization’s Problem Management Plus (PM+) (26). In Healthy Roads, CHWs aboard the mobile clinic deliver core PM+ skills, framed as wellness support to increase accessibility. The adaptation preserved fidelity to core PM+ components while adapting its language, framing, and integration with social service referrals to reflect the cultural and structural realities of Boston communities. Program refinement occurred through intentional feedback loops among participants, CHWs, supervisors, and community partners.

### 3.1 Identifying the need for a community-based mental health intervention

The Family Van (TFV) launched Healthy Roads, in January 2021 to provide medically underserved populations with free, culturally-relevant, linguistically-responsive mental health care. This program builds on decades of community engagement and long-term relationships with community leaders, community health centers, and faith-based organizations alongside global lessons from Partners In Health’s implementation across five countries since 2016.

CHW observed unmet mental health needs in TFV’s catchment neighborhoods. To better understand barriers to care and stressors, TFV conducted community listening sessions throughout 2018. Stakeholders highlighted structural barriers, including limited access to care; lack of racial, ethnic, and linguistic concordance between patients and providers; long wait times; insurance status; and immigration barriers. Stigma and mistrust of the mental health system were repeatedly cited as deterrents, even when services were available.

Community and client feedback highlighted need for a mental health intervention in a non-clinical setting, delivered by trusted community members, and addressing social determinants of health. Reported stressors included housing instability, financial strain, caregiving responsibilities, immigration concerns, and exposure to violence. Community feedback emphasized that emotional distress was inseparable from these social needs, underscoring the need to integrate mental health support with resource navigation and referrals. CHWs, trained to address specific burdens of each neighborhood, are positioned to deliver this contextualized model of care.

### 3.2 Core problem management plus (PM+) intervention delivered through healthy roads

PM+ is a transdiagnostic intervention that focuses on building four core skill areas.

- Stress management and grounding techniques.
- Structured problem-solving around client-identified life challenges.
- Behavioral activation to increase engagement in meaningful and routine activities.
- Strengthening and mobilizing social support.

Healthy Roads participants typically complete five individualized sessions, with flexibility to complete four to six sessions depending on need and progress. Adapted from the WHO PM+ manual, which is structured as five 90-min sessions, Healthy Roads sessions are generally 45–60 min to align with participants’ real-world constraints and can take place on the mobile clinic, in nearby community locations, or by phone or video as needed. Alongside PM+ skill-building, CHWs routinely provide warm handoffs and referrals to community resources and longer-term care when appropriate. This integration of coping skills with concrete resource navigation is a core component of Healthy Roads.

PM + integrates four core strategies, including managing stress, problem solving, behavioral activation, and strengthening social support in an iterative, skills-based format. These components build practical coping skills and are designed for delivery by trained non-specialist providers.

### 3.3 Choosing and adapting problem management plus (PM+) for healthy roads

In 2019, following listening sessions identifying gaps in mental health access, TFV, in consultation with Partners in Health (PIH), identified PM+ as an accessible intervention which could be piloted for community-based mental health support in its catchment areas. Program selection was shaped by the lived experience and expertise of people of color in Boston, alongside evidence-informed best practices.

**Figure 2:**
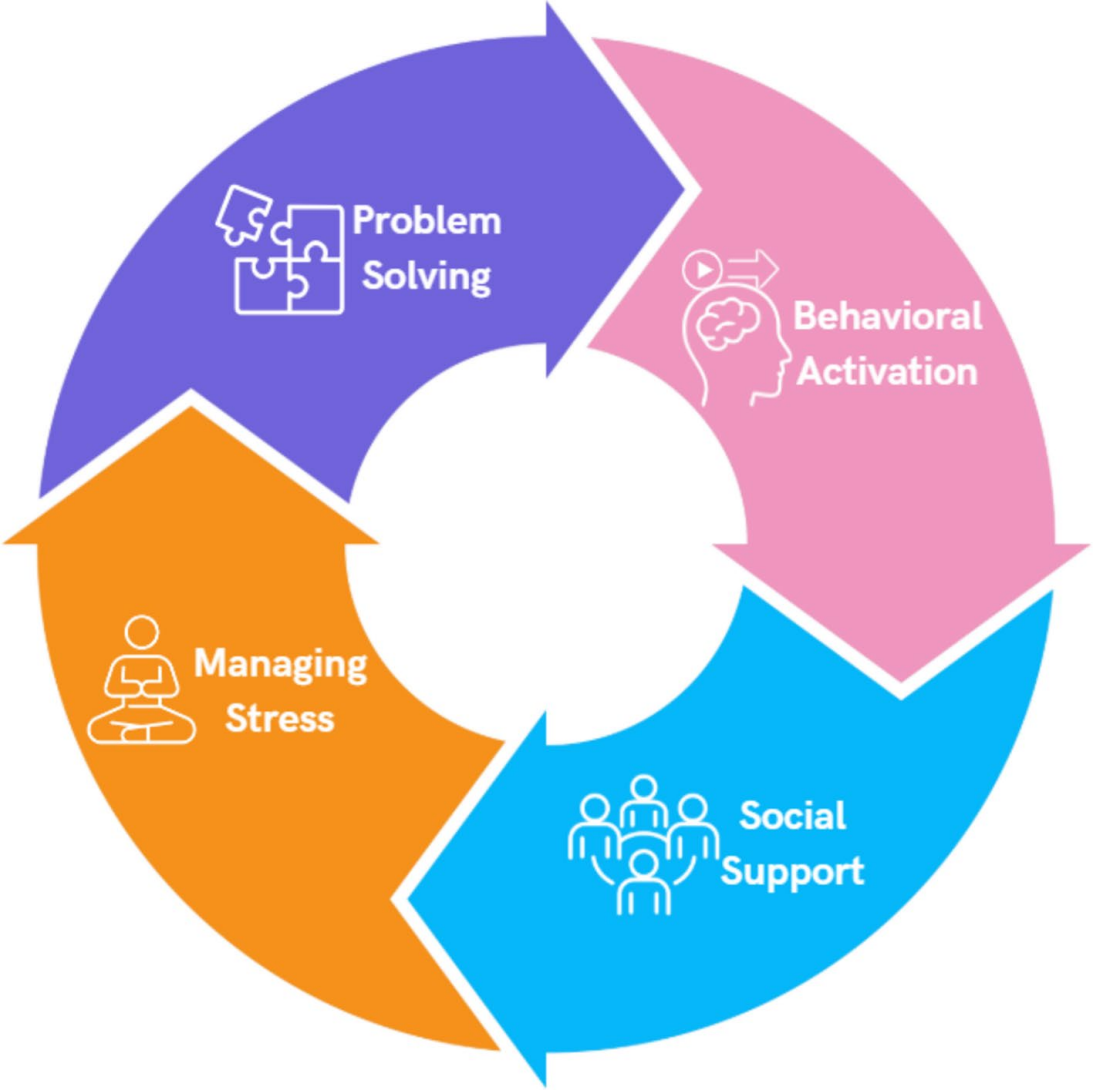
Core skills of Problem Management Plus (PM+), including stress management, structured problem-solving, behavioral activation, and strengthening social support. These evidence-based skills form the foundation of Healthy Roads and are delivered by trained community health workers.

TFV partnered with PIH to guide adaptation, implementation planning, and initial training. The adaptation process followed a structured, iterative approach informed by implementation science principles and PIH’s global experience in adapting structured mental health interventions, including (1) assessing community needs through listening sessions and CHW feedback, (2) preparing the PM+ intervention for a US community-based setting, (3) adapting training materials to local contexts, and (4) developing a care pathway (Figure 2) which articulated intervention entry and exit based on iterative models used across the PIH global sites, and (5) refining implementation through pilot delivery (28, 29). Adaptations included revising participant materials to align with community contexts, identifying appropriate screening and outcome tools, creating culturally responsive outreach materials, and integrating PM + with local social service referrals. These adaptations were iteratively refined based on CHW feedback and participant experience while maintaining core PM + components.

### 3.4 Training and supervising community health workers (CHWs)

PIH conducted initial PM+ training in fall of 2020 with select CHWs and a newly hired LICSW supervisor. Initial trainers included two PIH psychiatrists, a clinical psychologist, and a training coordinator. Due to the COVID-19 pandemic, training was conducted virtually through asynchronous learning modules and live skills-based sessions. The training spanned 25 h over 6 weeks and covered mental health in Massachusetts and globally; PM+ background and history; core competencies (helping skills, stress management, structured problem-solving, behavioral activation, strengthening social support); responding to emergencies; and ethical considerations such as confidentiality and boundaries. Training techniques included didactics, case presentations, skill demonstrations, and role play exercises with feedback to build practical delivery skills.

Following training, CHWs participated in ongoing supervision with the LICSW to support program fidelity, competency development, and care quality. Supervision also created a structured space for collective reflection and shared learning, where CHWs and supervisors collaboratively identified implementation challenges, adapted engagement strategies, and discussed evolving community needs.

During initial implementation, CHWs received weekly individual and monthly group supervision; individual sessions later shifted to bi-weekly. Supervision included case review discussions, skill reinforcement, troubleshooting challenging client scenarios, and CHW wellbeing support. All four CHWs participated in launching Healthy Roads, though three ultimately delivered sessions due to capacity constraints and integration into existing workloads. Over time, responsibilities were adjusted based on individual capacity, readiness, and role fit.

### 3.5 Care pathway and client flow

**Figure 3:**
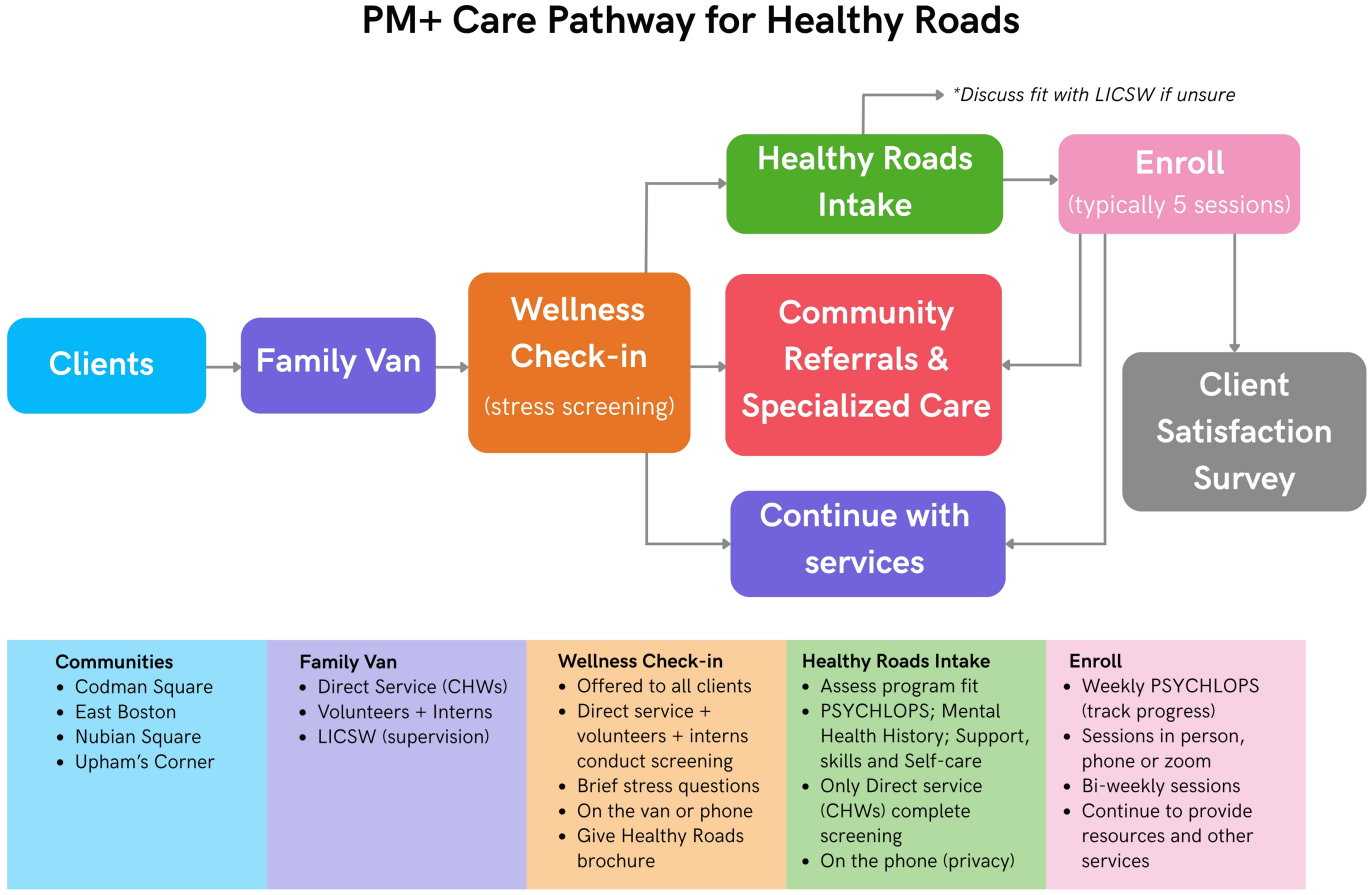
Healthy Roads Problem Management Plus (PM+) care pathway illustrating participant engagement, intervention delivery, and referral pathways. The pathway reflects the flexible, community-based implementation used by The Family Van.

Figure 3 illustrates the Healthy Roads care pathway. Clients typically enter the program through a conversational wellness check-in on the mobile clinic, then complete an intake to assess fit for PM+. Participants engage in a personalized series of PM+ sessions and receive referrals for social or specialized care as needed.

The pathway intentionally reflects the nonlinear and adaptive nature of implementing PM+ in a community setting. Clients are assessed for readiness and encouraged to complete all five sessions, while maintaining flexibility to accommodate real-world circumstances. Clients may enter Healthy Roads, pause, re-engage, or receive one-time support without being required to complete all sessions consecutively.

### 3.6 Program delivery and outreach

Early in the pilot implementation phase, Healthy Roads was shared as a mental health promotion program. CHWs observed that this language deterred some potential participants due to mental health stigma. In response to community feedback, Healthy Roads was then re-framed as a wellness support program grounded in “head-to-toe wellness.” This framing emphasized stress management, problem-solving, and overall wellbeing rather than diagnosis or treatment of mental illness. Outreach occurred directly through mobile clinic encounters, word of mouth, referrals from community partners, and both print and digital materials. All materials were designed to be culturally and linguistically responsive and to normalize stress as a common response to life challenges.

**Figure 4:**
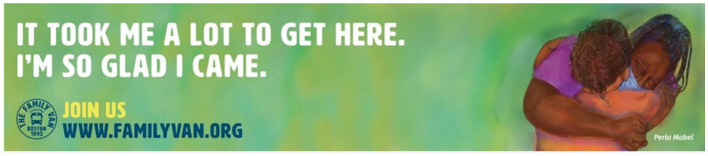
Example of a community outreach campaign developed for healthy roads.

To address stigma at a broader level, TFV launched a community art and communications campaign for Healthy Roads (Figure 4). Community advisory councils collaborated with local artists to co-create messages that promote mental wellbeing and invite conversation. The campaign was designed to spark intergenerational dialogue about mental health and wellbeing, reduce mental health stigma, and increase awareness of community-based mental health resources, with particular focus on Roxbury and Boston residents who identify as Latinx. Artwork and slogans developed through this process were displayed on community public bus lines, displayed in the mobile clinic, shared through social media, and distributed throughout neighborhood spaces.

## 4 Methods

### 4.1 Study design, setting, and implementation period

This mixed-methods community case study shares real-world implementation data to examine the novel pilot adaptation of PM+ in Boston. This study also documents the iterative co-learning processes that emerged throughout implementation and informed ongoing program refinement. By integrating program implementation insights, quantitative assessment of early participant outcomes, and qualitative insights from participant experiences, this study offers a practice-based understanding of how PM+ was adapted and delivered within a trusted mobile health clinic during the initial implementation period from January 2021 to December 2023. This period, spanning the COVID-19 pandemic and its aftermath, also offers valuable insight into how community-based mental health support can be sustained during a time of heightened social and economic stress and service disruption.

### 4.2 Participant identification, recruitment, and data collection

Participants were identified and recruited through routine service delivery on the mobile clinic, including regular preventive health screenings, CHW referrals, and referrals from community partners. Individuals who expressed interest in receiving mental wellness support or were identified by CHWs and volunteers as experiencing distress were invited to participate in the program. A total of 49 participants engaged in the program during the evaluation period and were included in analysis.

Participants’ health and demographic information were collected by the CHWs using survey questions. CHWs also collected data on client wellbeing and problems using baseline, weekly, and post-intervention Psychological Outcomes Profiles (PSYCHLOPS) scores (12). PSYCHLOPs is a brief, mental health outcome measure that gathers client-identified problems and associated distress, which was used in the initial effectiveness studies of PM+ (30). It was selected for this program because it allows participants to define their own primary concerns and offers a quantitative scale to measure the impact of this intervention (31).

### 4.3 Data collection and analysis

Quantitative data were analyzed descriptively to examine changes in psychological distress over time. Change in distress was defined as the difference between baseline and post-intervention PSYCHLOPS scores, and percentage reduction was calculated as the relative change from baseline to post-intervention at the individual level among participants with available follow-up data.

To explore the relationship between engagement in Healthy Roads and changes in distress, linear regression was used with change in PSYCHLOPS score as the outcome and number of sessions attended as the primary independent variable, adjusting for age, gender, and race. Analyses were conducted in R, with 95% confidence intervals estimated using likelihood-based methods and statistical significance defined as *p* < 0.05. As part of a novel, community-based pilot, analyses intentionally prioritized exploratory learnings to inform early-stage program refinement. All participants were included regardless of program completion, allowing for understanding of distress across the full range of community participation.

Qualitative data about clients’ stressors and experiences were gathered by CHWs during weekly sessions. Clients responded to open-ended questions at each session and completed a satisfaction survey at program completion. Data were reviewed using an iterative, inductive approach to identify themes in clients’ experiences, mental health concerns, and perceived program impacts. Themes were identified and refined through team review and discussion. Qualitative analysis focused on capturing key experiential insights to inform ongoing program refinement in a practice-based context.

### 4.4 Iterative program refinement

Continuous feedback from participants and CHWs informed ongoing refinements to outreach, session pacing, language, and referral partnerships throughout 2021–2023. Quantitative and qualitative findings were integrated to inform program refinement and to offer a holistic understanding of implementation and participant experiences.

## 5 Implementation outcomes

From January 2021 to December 2023, we conducted 173 sessions with 49 people. 51% of sessions were conducted in English, 41% in Spanish, and 4% in Portuguese. Participants reflected the diversity of communities served, with 32% identifying as Black, 54% Latinx, 8% White, and 6% without race information. In addition, 50% of participants were covered by public insurance, reflecting the program’s reach among lower-income individuals (Table 1).

**Table 1:** Characteristics of clients enrolled in healthy roads, January 2021–December 2023.

Characteristic	Number	Percentage
Gender		
Female	35	71.4
Male	14	28.5
Race		
Black or African American	16	30.1
Latinx or Hispanic	27	50.9
White or Caucasian	4	7.5
Prefer not to answer	3	5.6
Ethnicity		
African	1	2.5
Black or African American	13	32.5
Cape Verdean	13	32.5
Caribbean Islander	2	5.0
Colombian	2	5.0
Dominican	1	2.5
European	2	5.0
Haitian	1	2.5
Honduran	5	12.5
Age		
20–29	2	4.08
30–39	6	12.24
40–49	6	12.24
50–59	17	34.69
60–69	9	18.37
70–79	8	16.33
>80	1	2.04
Primary language		
English	25	50.00
Spanish	20	40.00
Portuguese	2	4.00
Haitian Creole	1	2.00
Prefer not to answer	1	2.00

Thirty percent of clients at TFV screened positive for mental health distress, and 72.1% of eligible clients enrolled in Healthy Roads. Additionally, 362 community-based referrals for social and emotional support were made through the program. The higher number of referrals reflects high level of unmet need identified during screenings whereas enrollment required readiness for a multi-session intervention. Staff noted that building readiness for PM + often required multiple outreach and relationship-building interactions over time. Clients who completed the program (e.g., participated in all 5 sessions) experienced an average 25.4% reduction in mental health distress 95% CI [13.1, 37.9%]. This estimate reflects the mean relative change in PSYCHLOPS scores from baseline to post-intervention among participants with available follow-up data.

**Figure 5:**
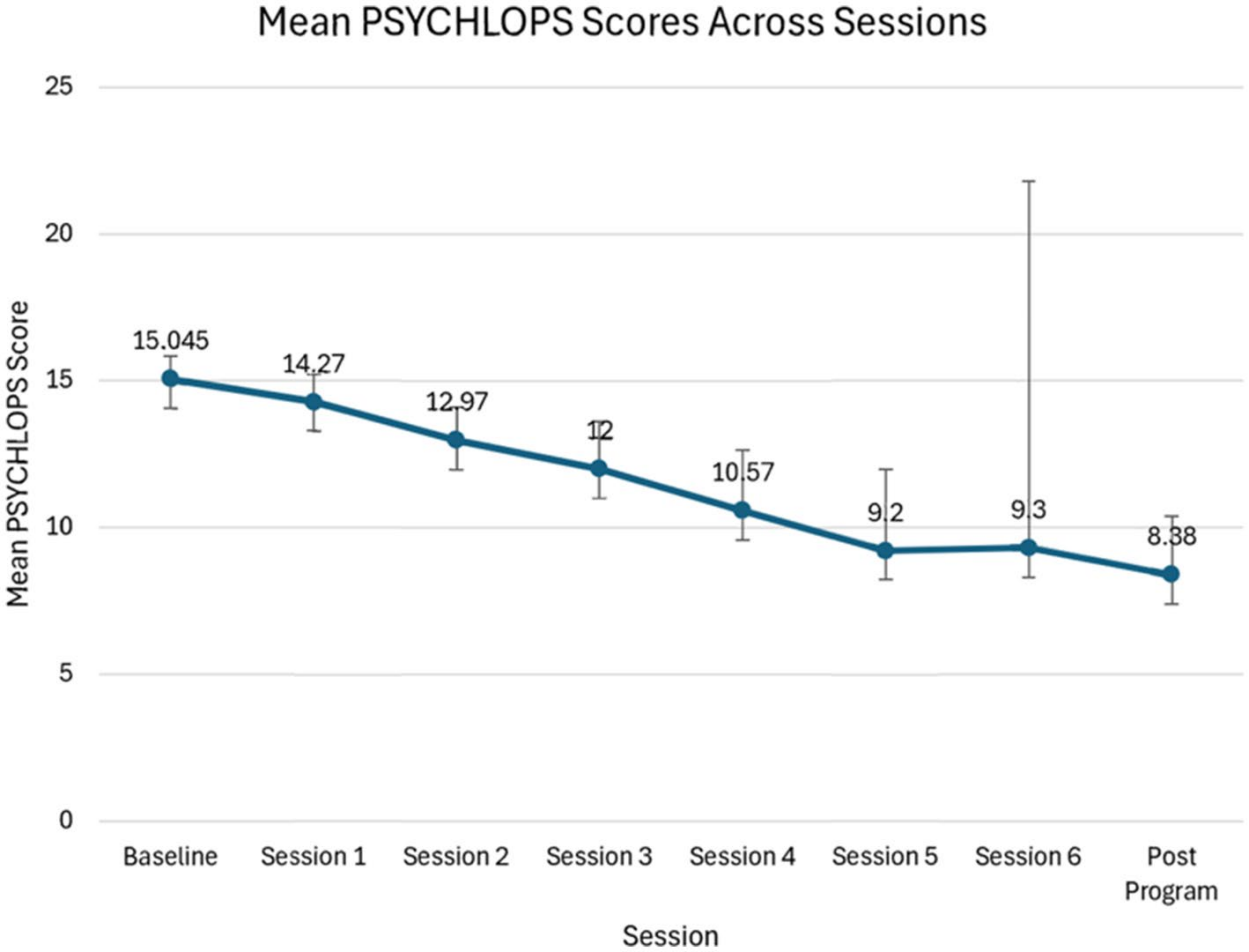
Mean PSYCHLOPS scores across healthy roads sessions. Early program outcomes show mean PSYCHLOPS scores decreased from baseline through subsequent sessions, reflecting reductions in self-reported distress among participants. Error bars represent 95% confidence intervals. Sample sizes varied across sessions due to the flexible and adaptive nature of program participation. Fewer participants at later sessions, particularly session six, contributed to wider confidence intervals.

Clients who engaged in at least one session experienced an average 11.4% reduction in mental health distress 95% CI [5.8, 17.0%]. There was also a statistically significant relationship between the number of sessions attended and changes in distress scores (*β* = −1.2, *p* < 0.05), indicating that each additional session was associated with an average decrease in PSYCHLOPS score. This corresponds to an approximate 7.5% reduction in distress per additional session (Figure 5).

## 6 Discussion

This community case study presents a novel implementation of Problem Management Plus (PM+) in a community health worker-led mobile clinic in a high-income, community setting. Healthy Roads offers proof-of-concept of a low-intensity, evidence-based protocol delivered through a unique health care model. Insights from Healthy Roads demonstrate how PM + can be adapted for settings characterized by structural inequities, workforce shortages, and fragmented care. Implementation required ongoing adaptation, staff support, and community trust. Participants reported reductions in distress and increased engagement with support services. These exploratory findings suggest the potential of this community-based model to increase access to mental health support. These adaptations were part of an intentional process of learning from community experience and translating lessons into evolving program practices, creating a dynamic system of collaborative knowledge generation.

These findings contribute to a growing body of literature on task-shifting and community-based mental health interventions, showing that non-specialist delivery models can increase access to care, expand workforce capacity, and reduce psychological distress across diverse settings. Insights support prior studies of PM + which have shown effectiveness in both low- and middle-income countries and, more recently, feasibility in high-income settings, particularly among populations experiencing structural barriers to care (23, 32). While most studies are trial-based, this pilot contributes practice-based insights on adapting and delivering PM + in a community-driven, non-clinical setting. These findings are consistent with implementation science frameworks, which emphasize feasibility, acceptability, and adaptability as core early-stage outcomes for novel interventions (33).

Implementation highlighted the importance of flexible care pathways. Clients often moved in and out of support depending on readiness, life circumstances, and connection to CHWs or volunteers. In response, TFV developed additional PM + -informed resources for clients seeking one-time or brief support. Healthy Roads expanded to train other community-based organizations across Massachusetts, completing a three-year cohort with five organizations and preparing a new two-year cohort. Healthy Roads illustrates how community-based mental health implementation contributes to health systems learning. Lessons generated through participant engagement, CHW supervision, program adaptation, and cross-organizational collaboration informed training and expansion with other organizations. Implementation knowledge not only contributed to internal program improvement but also to a broader ecosystem of shared learning about community-centered mental health delivery.

### 6.1 Key insight 1: community health workers are essential for providing culturally responsive care

CHWs played a critical role in helping clients address stressors related to the social determinants of health. Participant feedback and program observations indicated that CHWs facilitated connections to community resources, provided practical support such as identifying affordable housing, and empowered clients with skills to manage stressors on their own terms. By addressing root causes alongside mental health symptoms, CHWs provided comprehensive support. These findings reflect task-shifting literature showing that non-specialist providers expand access to care and improve engagement, particularly in settings with workforce shortages and limited access to culturally responsive mental health services (34, 35).

In addition, cultural concordance and trust between CHWs and clients facilitated engagement. CHWs are uniquely positioned to provide mental health support that aligns with community needs and to shape social perceptions of mental health. CHWs drew on their lived experience to connect with clients. For instance, one client described how she felt supported when her CHW incorporated prayer into sessions based on shared religious beliefs: “She integrated the spiritual aspect which really helped me, and she used affirmations which helped build my self-esteem”.

### 6.2 Key insight 2: relational, client-led approaches enhance trust and improve participation

Program experience suggested that a relational, client-led approach better captured participants’ distress than standardized screening tools alone. During the first 6 months, the program used common evaluation tools including the PHQ-9 and GAD-7, administered at baseline and post-intervention. However, clients disclosed that these approaches felt impersonal or difficult to understand. Participant feedback also indicated that mental health stigma remained a barrier to engagement. In response, the program transitioned to conversational wellness check-ins using open-ended questions and client-led conversations. The program also incorporated the PSYCHLOPS as a validated, patient-centered outcome measure designed to capture changes in mental wellbeing beyond symptom-focused screening by centering priorities most important to clients. One client noted: “My needs were understood and acknowledged and using a holistic view of me as a person, I was given coping tools and strategies that worked for me.” Framing Healthy Roads as wellness support rather than formal treatment appeared to reduce stigma and improve engagement.

Participants also appreciated Healthy Roads’ client-led approach, which integrates clients’ own vocabulary and goals to guide each session. Client’s perceived improvements in their skills to manage future stressors, felt decreased immediate distress, and progressed on goals surrounding employment, connecting to long term care, and housing stability. One client described Healthy Roads as “a good entry point” into longer-term support. Overall, clients expressed an improved sense of wellbeing. Staff also learned that enrollment often required multiple conversations with clients and community partners, sometimes over weeks or months. This process highlighted the importance of intentional framing and sustained relationship-building.

### 6.3 Key insight 3: flexibility in implementation is crucial for engagement

Early implementation showed that the standard PM + structure of five weekly 90-min sessions did not align with all clients’ schedules or daily realities. Participants often balanced multiple jobs, caregiving responsibilities, unstable housing, and transportation barriers. In response, CHWs adapted the program to five 60-min sessions based on client availability, with the typical cadence every other week. While clients were encouraged to complete all five sessions, progress made in fewer or shorter sessions was still recognized, and some clients completed an additional sixth session when needed.

Rather than enforcing a rigid timeline or frequency of sessions, CHWs adjusted session length, cadence, and total number of sessions on a case-by-case basis while retaining core PM + skills. Flexibility improved client retention, satisfaction, and uptake of coping and problem-solving strategies. Tailoring delivery to structural constraints enabled meaningful engagement with the intervention.

### 6.4 Key insight 4: hybrid delivery models can expand access

Hybrid phone or video formats alongside in-person delivery expanded reach and reduced transportation, work, caregiving, and privacy barriers. The COVID-19 pandemic led to unexpected challenges and opportunities in providing mental health support to communities already experiencing significant barriers to care. As a result of pandemic safety measures, the program launched in a virtual format over the phone or Zoom. Due to limited in-person outreach and meetings, community interest in the program spread primarily through word-of-mouth during the first 6 months. After a return to in-person work, the program continued in a hybrid format, which increased accessibility for clients.

While initially designed for delivery on the mobile clinic, the pandemic prompted the development of a more flexible workflow, with screenings conducted on the van and sessions occurring in trusted community spaces or through telehealth. This approach appeared to better balance in-person engagement and trust building with the need for privacy and accessibility. This hybrid model supported continuity of services for our clients and helped address common barriers to care including transportation, caregiving, work schedules, and privacy concerns.

### 6.5 Key insight 5: integrating social determinants of health into mental health support increases impact

Healthy Roads demonstrated that psychological distress among clients was inseparable from structural issues such as housing instability, food insecurity, unemployment, trauma, or financial strain. Some clients described these challenges as survival problems to endure and did not initially identify them as affecting their mental health even when they were experiencing significant emotional distress. CHWs, who routinely provided wraparound services on the mobile clinic, integrated PM + skills with resource referral and support with social determinants of health. CHWs made community-based referrals for housing applications, financial assistance, food resources, domestic violence support, and long-term therapy. This dual focus helped clients reframe overwhelming life challenges as both practical problems that could be addressed and legitimate sources of emotional strain worthy of support.

Clients reported relief when CHWs addressed concrete social stressors alongside emotional coping. Client feedback suggests PM + becomes more impactful when coupled with housing, food, financial, and safety support as mental health cannot be separated from the structural inequities shaping it. Participant experiences highlighted the close relationship between social determinants of health and psychological distress, suggesting that integrating wraparound support with mental health support may enhance relevance and engagement. In addition, TFV’s longstanding community partnerships give our CHWs insight into accessible and appropriate referrals. Organizations seeking to replicate this model may need to invest significant time in relationship-building and community engagement to develop similar referral networks.

### 6.6 Key insight 6: community-led program framing is essential for engagement

Program uptake depended not only on service availability but also how the intervention was described and presented. Mental health stigma remains a barrier to care, and reframing Healthy Roads as wellness support based on community feedback reduced stigma-related resistance and increased engagement. This shift suggests that access to mental health services is tied to language, symbolism, and cultural meaning.

Community-designed art and messaging extended these conversations about mental wellbeing into community shared spaces. Pairing evidence-based psychological support with community-led cultural expression helped normalize help-seeking and position mental wellbeing as a collective concern.

### 6.7 Key insight 7: staff support and supervision are essential for program sustainability

Delivering mental health support requires wellness skills, boundary management, and burnout prevention. Mental health care providers are at particular risk for burnout as actively listening to someone else’s challenges and helping them develop resilience requires emotional labor (36, 37). CHWs were offered bi-weekly group and individual supervision, workload adjustment, professional development, and formal team building. Staff also had regular opportunities to practice self-care during working hours and to connect with colleagues. Redistributing roles also ensured sustainable workloads for staff. Implementation experience highlighted the importance of staff wellbeing, caseload management, reflective supervision, professional development, and organizational support to prevent burnout.

## 7 Limitations

This community case study of the Healthy Roads program has several limitations. As an early, innovative implementation in a dynamic community context, this study prioritized rapid, iterative learning and responsiveness to community needs. Future studies should build on these findings to examine scalability and causal impact in large, multi-site samples. The flexibility and adaptations that allow Healthy Roads to address diverse client needs also introduce challenges for standardized evaluation. Definitions of success varied across clients, complicating standard evaluation. The program was implemented within a single mobile clinic program with a relatively small sample size, which may limit generalizability.

While PSYCHLOPS was a useful measure for many clients, not all participants completed post-intervention assessments due to life events such as employment changes, relocation, or health needs. In some cases, participants reported learning valuable skills or feeling supported without measurable reductions in distress. For others, the initial conversation represented a meaningful first step toward disclosure or connection to longer-term care. The absence of a control group limits attribution of changes in distress exclusively to the intervention. Although PSYCHLOPS is validated in diverse settings, its use within the U.S. among racially and linguistically diverse settings require further evaluation.

The limited amount of qualitative data reflects an intentional effort to uphold confidentiality, increase trust and engagement while minimizing the burden of data collection for both clients and CHWs. This approach respects the autonomy of program participants but may limit the depth of captured experiences. Programs seeking to replicate this model should account for the time and resources required to build trust, support CHWs, and adapt interventions to local context, which are elements that require sustained investment to scale.

## Data Availability

The raw data supporting the conclusions of this article will be made available by the authors, without undue reservation.
